# Biocompatible Films of Calcium Alginate Inactivate Enveloped Viruses Such as SARS-CoV-2

**DOI:** 10.3390/polym14071483

**Published:** 2022-04-06

**Authors:** Alba Cano-Vicent, Rina Hashimoto, Kazuo Takayama, Ángel Serrano-Aroca

**Affiliations:** 1 Biomaterials and Bioengineering Laboratory, Centro de Investigación Traslacional San Alberto Magno, Universidad Católica de Valencia San Vicente Mártir, c/Guillem de Castro 94, 46001 Valencia, Spain; alba.cano@mail.ucv.es; 2 Center for iPS Cell Research and Application (CiRA), Kyoto University, Kyoto 606-8507, Japan; rina.hashimoto@cira.kyoto-u.ac.jp

**Keywords:** calcium alginate, SARS-CoV-2, bacteriophage, phi 6, biomaterials, films, hydrogels

## Abstract

The current pandemic is urgently demanding the development of alternative materials capable of inactivating the severe acute respiratory syndrome coronavirus 2 (SARS-CoV-2) that causes the coronavirus 2019 (COVID-19) disease. Calcium alginate is a crosslinked hydrophilic biopolymer with an immense range of biomedical applications due to its excellent chemical, physical, and biological properties. In this study, the cytotoxicity and antiviral activity of calcium alginate in the form of films were studied. The results showed that these films, prepared by solvent casting and subsequent crosslinking with calcium cations, are biocompatible in human keratinocytes and are capable of inactivating enveloped viruses such as bacteriophage phi 6 with a 1.43-log reduction (94.92% viral inactivation) and SARS-CoV-2 Delta variant with a 1.64-log reduction (96.94% viral inactivation) in virus titers. The antiviral activity of these calcium alginate films can be attributed to its compacted negative charges that may bind to viral envelopes inactivating membrane receptors.

## 1. Introduction

The COVID-19 pandemic has forced material engineers to develop and search for alternative materials capable of inactivating SARS-CoV-2 [1,2,3,4,5,6,7,8]. In this regard, sodium alginate (SA) has shown antiviral activity against several types of viruses [9] and has been seen to possess excellent chemical, physical, and biological properties that render this biopolymer very useful for an immense range of industrial applications [10,11,12,13,14,15,16,17]. This carbohydrate polymer presents a linear structure of (1–4)-linkedβ-D-mannuronic acid (M) blocks and α-L-guluronic acid (G) blocks, which are arranged in a block wise fashion with M and G blocks in different ratios and sequences depending on the type of alginate [18,19,20]. This hydrophilic material is biodegradable, biocompatible, non-toxic, and economic in comparison with other biopolymers, and renewable because it can be produced from brown algae or microbial culture sources [21]. SA is a water-soluble polymer that can be crosslinked with salts containing divalent cations such as calcium chloride to form hydrogels, that is, polymer gels that can absorb large amounts of water without being dissolved [22]. The gelation process occurs when divalent cations of Ca^2+^ interact with G blocks according to the egg-box model buckled structure [23]. The antiviral activity of SA has shown to increase with increasing the amount of G blocks presents in the backbone polymer structure [24]. However, the antiviral activity of calcium alginate against enveloped viruses has been hardly studied [25,26]. These previous antiviral studies of calcium alginate against enveloped viruses, such as Influenza (IFV), Hepatitis C virus (HCV), and Sindbis virus (SINV), showed low or negligible antiviral activity. However, the calcium alginate tested against these viruses were fabricated in the form of fibers or microcapsules. Thus, as the antiviral mechanism of action of SA and calcium alginate seems to be associated with the negative charges of the anionic biopolymer that can bind to viral envelopes [9], we hypothesize here that calcium alginate in the form of films synthesized by a particular procedure able to concentrate its negatively-charged polymer chains will be able to inactivate enveloped viruses such as SARS-CoV-2 Delta variant and bacteriophage phi 6. The bacteriophage phi 6 is often used as surrogate of SARS-CoV-2 [27] due to the fact that it is an enveloped virus that can be handled in safe conditions [28]. The pathogenic SARS-CoV-2 Delta variant and the new variants that are emerging, such as omicron sub-lineage BA.2, are a great risk to global public health [29]. The SARS-CoV-2 Delta variant is highly transmissible and possesses mutations that may partially confer immune escape [30]. In fact, outbreak investigations suggested that vaccinated people can spread SARS-CoV-2 Delta in a highly vaccinated country [31,32]. The toxicological aspects of the synthesized calcium alginate films were analyzed in human keratinocytes HaCaT cells to study if these films could be used for biomedical applications.

## 2. Materials and Methods

### 2.1. Materials

Sodium alginate and calcium chloride (anhydrous, granular, ≤7.0 mm, ≥93.0%) was purchased from Sigma-Aldrich (Saint Louis, MO, USA).

The SA used in this study was characterized by ^1^H-NMR spectroscopy. Thus, the SA sample was hydrolyzed to an average chain length of 30–50 sugar units, followed by freeze drying. Lyophilized samples (8–10 mg) were dissolved in D_2_O to a final volume of 600 µL D_2_O (99.9% Sigma-Aldrich, Saint Louis, MO, USA). 3-(Trimethylsilyl)-propionic-2,2,3,3-d4 acid sodium salt (TSP) (Aldrich, Milwaukee, Brookfield, WI, USA) in D_2_O (1%, 2.5 µL) was added for internal chemical shift reference. ^1^H-NMR spectra were recorded at 82 °C on a BRUKER AVIIIHD 400 MHz equipped with 5 mm SmartProbe 1D. The spectra were recorded using TopSpin 3.2 or 3.5 software (Bruker BioSpin, Ettlingen, Germany) and processed and analyzed with TopSpin 4.0.6 software (Bruker BioSpin). The ASTM protocol was used for ^1^H NMR characterization [33]. The results of this analysis show that G and M blocks as F_G_ = 0.463, F_M_ = 0.537, or M/G ratio of 1.16, F_GG_ = 0.282, F_GM_ = F_MG_ = 0.181, F_MM_ = 0.356, F_GGM_ = F_MGG_ = 0.048, and F_GGG_ = 0.234. The guluronic acid blocks are mostly arranged in short G-blocks (average length ~7).

### 2.2. Film Preparation

First, 0.25 g of SA was dissolved in 30 mL of distilled water by magnetic stirring at 24 ± 0.5 °C for 1 h. This mixture was poured into a circular mold and dried to form films by solvent evaporation. Thus, the mold was left at room temperature (24 ± 0.5 °C) under an extraction hood for 24 h, followed by 48 h at 37 °C in an oven. After that, the films were crosslinked by immersion for 1 h in a crosslinking solution prepared by dissolving 5 g of calcium chloride in 500 mL of distilled water (1% *w*/*v*) [34] under magnetic stirring for 15 min at 24 ± 0.5 °C. After crosslinking the film, it was washed in distilled water three times to ensure complete removal of calcium chloride residues and left to dry for 24 h at room temperature, followed by 48 h at 37 °C. The films were cut into disk specimens of 10 mm diameter and sterilized by ultraviolet radiation 1 h per side. This sample is hereafter referred to as AlgCa.

### 2.3. Toxicological Study

The films were introduced in a six-well plate using Dulbecco’s Modified Eagle’s Medium (DMEM, Biowest SAS, Nuaillé, France) without Fetal bovine serum (FBS) to fill the wells up to completely cover the surface area. These cytotoxicity tests were performed using a surface material/volume ration of 3 cm^2^/mL according to the ISO-10993 standard recommendations. The film disks were incubated in humidified 5% carbon dioxide/95% air ambient for 72 h at 37 °C to produce the extracts. Right after that, the extracts were collected and used for the cytotoxicity tests. The cell line used in the cytotoxicity assays was donated by the Biomedical Research Institute and Hospital La Fe, Valencia, Spain, and consisted of non-tumorigenic immortalized human keratinocyte HaCaT cells. Cell growth was carried out using DMEM with 10% FBS, 100 units/mL penicillin (Lonza, Verviers, Belgium), and 100 mg/mL streptomycin (HyClone, GE Healthcare Life Sciences), at a temperature of 37 °C and 5% carbon dioxide. The cytotoxic effects of the film extract on cell viability were analyzed by the 3-[4,5-dimethylthiazol-2-yl]-2,5-diphenyl tetrazolium bromide (MTT) method [35,36,37]. The human keratinocytes were planted at a density of 10^4^ cells/well onto a 96-well plate. Incubation of the cells was performed for 24 h at 37 °C before replacing the culture medium with 100 μL of film extract in each well. A negative control was measured, replacing the culture medium with 100 μL of the same medium used to produce the film extracts. A positive control was measured, replacing the culture medium with 100 μL of 1000 μM zinc chloride (≥97.0%, Sigma-Aldrich, Saint Louis, MO, USA) cytotoxic solution [38]. A mass/volume amount of 5 mg/mL MTT in each well was used to incubate the human cells for 3 h. Finally, the dissolution of the formazan crystals was performed in 100 μL of dimethyl sulfoxide (Sigma-Aldrich, Saint Louis, MO, USA) at 24 ± 0.5 °C to measure the absorbance 550 nm with the help of a microplate reader (Varioskan, Thermo Fisher, Waltham, MA, USA).

### 2.4. Antiviral Test with the Bacteriophage Phi 6

*Pseudomonas syringae* (DSM 21482) and bacteriophage phi 6 (DSM 21518) were purchased from the Leibniz Institute DSMZ-German Collection of Microorganisms and Cell Cultures GmbH (Braunschweig, Germany). This Gram-negative bacterium was cultured in solid tryptic soy agar (TSA, Liofilchem) and subsequently in liquid tryptic soy broth (TSB, Liofilchem) at 120 r.p.m. and a temperature of 25 °C. Bacteriophage phi 6 propagation was carried out according to the Leibniz Institute DSMZ-German Collection of Microorganisms and Cell Cultures GmbH specifications. In these antiviral assays, a bacteriophage suspension volume of 50 μL was added in TSB to each AlgCa film disk at a titer of about 1 × 10^6^ plaque-forming units per mL (PFU/mL) to be incubated for 30 min. The AlgCa disks were individually placed in falcon tubes with a TSB volume of 10 mL to be sonicated at 25 °C for 5 min, followed by 1 min vortexing. After that, serial dilutions were performed from each falcon tube for bacteriophage titration. A bacteriophage dilution volume of 100 μL was mixed with a host strain volume of 100 μL at OD_600nm_ = 0.5. Thus, the bacteriophage infective capacity was studied according to the double-layer assay [39]. A volume of 4 mL of top agar (TSB + 0.75% bacteriological agar) from Scharlau (Ferrosa, Barcelona, Spain) with 1 mM calcium chloride and the bacteriophage/bacteria suspension were mixed and poured on TSA plates to be cultured at 25 °C in a refrigerated incubator for 24 h. The antiviral activity was determined at 30 min of contact by calculating the bacteriophage titers in log(PFU/mL) for comparative analysis with the control sample, that is, a similar bacteriophage suspension volume of 50 μL mixed with the bacteria without having been in contact with the AlgCa film disk. The null effect of the sonication/vortexing processes on the infectious activity of the bacteriophage phi 6 and the absence of interference of the residual amounts of the calcium alginate films with the titration procedure were checked to avoid false results. These antiviral assays were performed three times in two different days (*n* = 6) to provide reliable results.

### 2.5. Bacteriophage RNA Extraction and Quantification

In order to ensure that the viral particles do not remain adhered to the AlgCa films providing false positive results in the antiviral experiments, double-stranded RNA extraction and quantification of the bacteriophage phi 6 were performed before and after being in contact with the calcium alginate films. These tests are very important to ensure that the viruses inactivate after being in contact with the AlgCa films. A volume of 50 μL with a bacteriophage concentration of 1 × 10^6^ PFU/mL was dispersed on the AlgCa film disks and left to incubate at 25 °C for 30 min. A volume of 50 μL of the same bacteriophage dispersion, without having been in contact with the films, was left to incubate at 25 °C for 30 min (control). After incubation, 10 mL of TSB were mixed with the samples to perform sonication and vortexing for 5 min and 45 s, respectively. After that, RNA extraction was carried out using the RNA extraction protocol of Norgen Biotek Corp. (Thorold, ON, Canada) [40]. Thus, viral particle-lysing was performed to produce a transparent mixture. After this first step of the protocol, RNA purification was performed by binding this molecule to the purification column, washing the purification column, and eluting the RNA for storage at −70 °C to avoid degradation. Finally, the amount of RNA was quantified with a nanodrop (Thermo Scientific, Waltham, MA, USA) to express the results in ng/μL. These extractions and quantifications were performed in triplicate to provide reproducible values.

**Figure 1:**
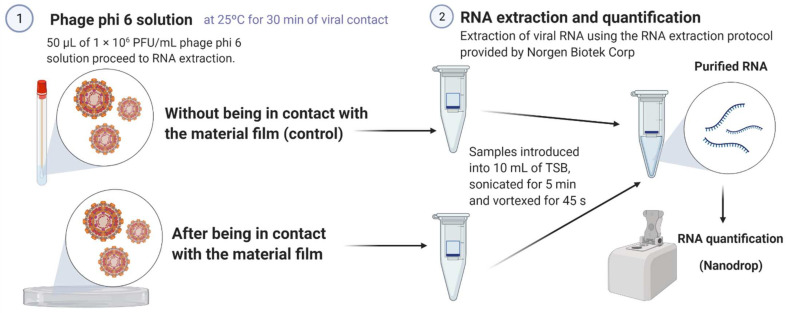
Bacteriophage phi 6 RNA extraction and quantification: Schematic representation of the RNA extraction and quantification process protocol to ensure that the viral particles do not remain attached to the material film instead of being inactivated. Created by Ángel Serrano-Aroca with Biorender.com.

Figure 1 shows a scheme that graphically summarizes the bacteriophage RNA extraction and quantification protocol used in these experiments.

### 2.6. Antiviral Test with SARS-CoV-2 Delta Variant

The SARS-CoV-2 strain (B.1.617.2, Delta) provided by BEI Resources (NR-55611, hCoV-19/USA/PHC658/2021) was stored at −80 °C. Plaque-purification and propagation was performed in TMPRSS2/Vero cells. A total of 500 μL of SARS-CoV-2 viral suspension in minimum essential medium (MEM, Sigma-Aldrich, Darmstadt, Germany) was added to each AlgCa film at 1.0 × 10^6^ median tissue culture infectious dose (TCID50) per film (TCID50/film), and then incubated at room temperature for 30 min. Then, 500 μL MEM were added to each AlgCa film and vortexed for 5 min. Viral titers were measured by the TCID50 assays in a Biosafety Level 3 laboratory at Kyoto University. Thus, TMPRSS2/Vero cells (JCRB1818, JCRB Cell Bank), cultured with MEM supplemented with 5% FBS and 1% penicillin/streptomycin, were seeded into 96-well plates (Thermo Fisher Scientific, Waltham, MA, USA). Serial dilutions 10-fold from 10^−1^ to 10^−8^ were performed to be placed onto the TMPRSS2/Vero cells in triplicate and incubated at 37 °C for 96 h. Microscopic evaluation of the cytopathic effects and the Reed–Muench method was used to determine TCID50/mL.

### 2.7. Statistical Analysis

One-way ANOVA with subsequent Tukey’s post hoc test and Student’s *t*-test was carried out for multiple and pair comparisons, respectively, using the GraphPad Prism 6 software.

## 3. Results

### 3.1. Toxicological Study

**Figure 2:**
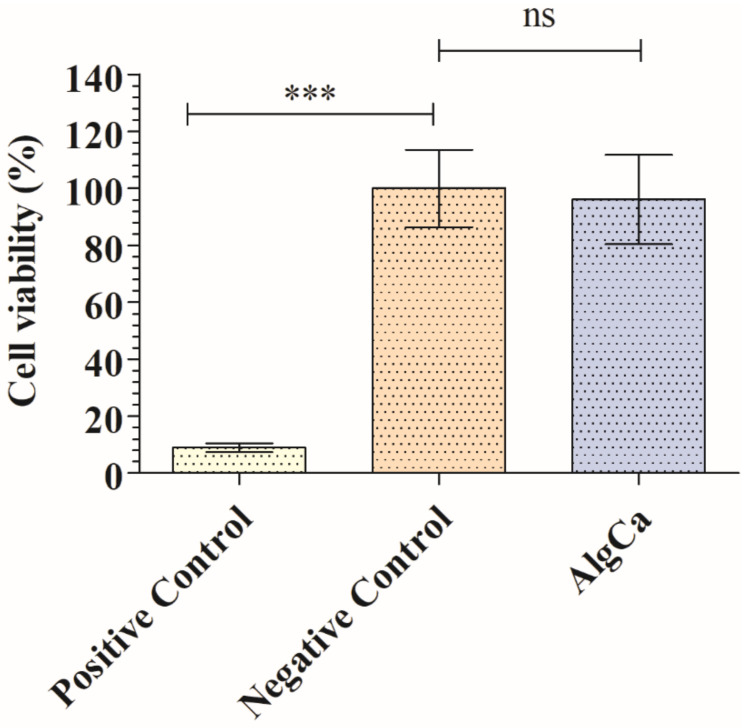
MTT cytotoxicity tests of extracts obtained from the calcium alginate films (AlgCa), negative control (culture medium used to produce the films extracts), and positive control (cytotoxic 1000 μM zinc chloride solution) cultured in human keratinocyte HaCaT cells at 37 °C. ANOVA with subsequent Tukey’s post hoc test: *** *p* > 0.001; ns, not significant.

The cytotoxicity of the calcium alginate film was studied in order to see if this material could be used in biomedical applications as antiviral agent. Figure 2 shows the percentages of the cell viability of the human keratinocytes HaCaT cultured in the AlgCa extract with respect to their culture in the cell growth medium (negative control, 100% cell viability).

Thus, the extracts of AlgCa films showed no cytotoxic effects in human keratinocytes. Thus, no statistically significant differences in cell viability (%) between the extract of the AlgCa film and the negative control was observed. The positive control of toxic solution significantly affected cell viability, as expected.

### 3.2. Antiviral Assays with the Enveloped Bacteriophage Phi 6

**Figure 3:**
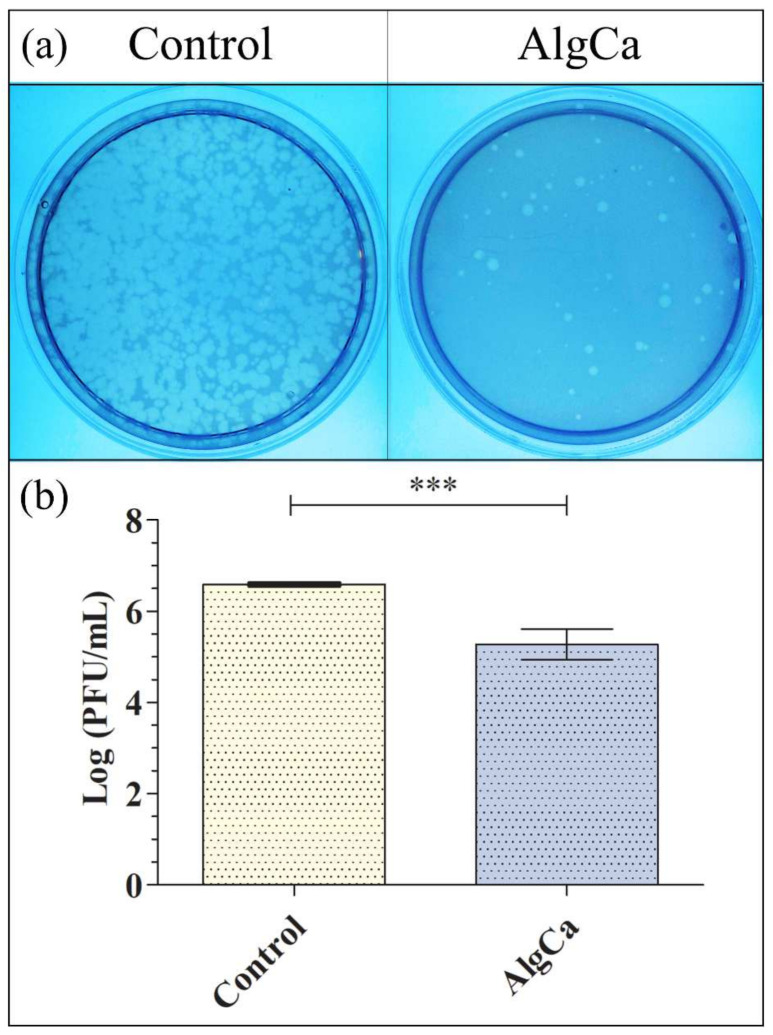
Antiviral activity of calcium alginate against bacteriophage phi 6 determined by the double-layer method: (**a**) Titration images (undiluted samples) for AlgCa film and control after 30 min of viral contact. These images show the reduction in infection capacity (reduction in lighter spots); (**b**) Viral inactivation in log (PFU/mL) for AlgCa film and control after 30 min of viral contact. Student’s *t*-test (*** *p* > 0.001).

Figure 3 shows the antiviral results obtained for bacteriophage phi 6 after being in contact with the AlgCa film in comparison with control, which is the same viral suspension mixed with the bacteria without having been in contact with the AlgCa film disk.

These results showed how the bacteriophages of the control sample are able to infect most bacterial cells (Figure 3a). However, the infective capacity of the bacteriophages after being in contact with the AlgCa film disk is reduced significantly (see reduction in lighter spots in Figure 3b). Thus, the synthesized calcium alginate film possesses antiviral activity against the bacteriophage phi 6 with a 1.43-log viral infectivity reduction (94.92% viral inactivation) after 30 min of viral contact.

### 3.3. Bacteriophage RNA Extraction and Quantification

**Figure 4:**
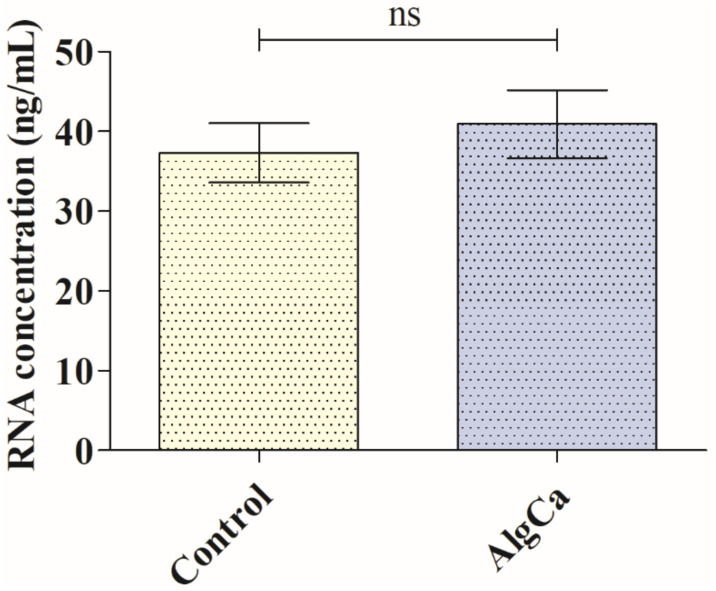
Bacteriophage phi 6 RNA extraction and quantification: Bacteriophage RNA concentration before (control) and after being in contact with the calcium alginate (AlgCa) films for 30 min expressed in ng/μL. Student’s *t*-test (ns, not significant).

Figure 3 shows the antiviral results obtained for bacteriophage phi 6 after being in contact with the AlgCa film in comparison with control. In order to make sure that the bacteriophages phi 6 does not remain attached in the inner part or the surface of the calcium alginate films before the antiviral assays, which would provide false results, RNA extraction and quantification of the bacteriophages were performed before and after being in contact with the material. Thus, Figure 4 shows similar amounts of bacteriophage RNA with no significant differences before (control) and after being in contact with the AlgCa films.

### 3.4. Antiviral Assays with the SARS-CoV-2 Delta Variant

**Figure 5:**
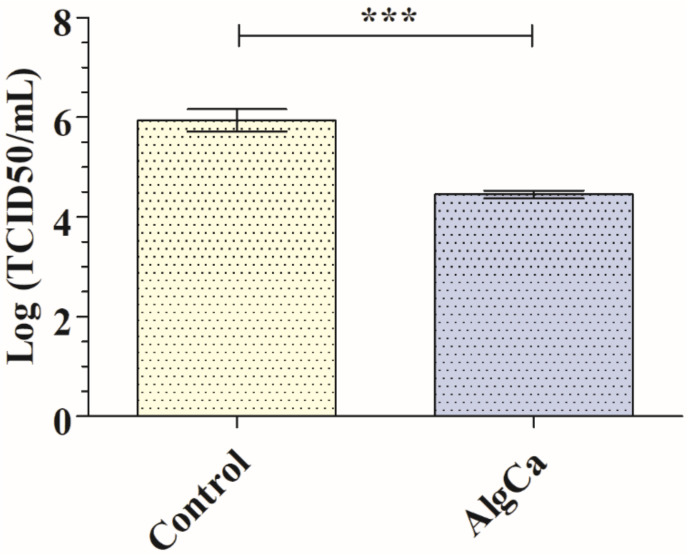
Reduction in infection titers of the SARS-CoV-2 Delta Variant in logarithm of TCID50 per mL (log (TCID50/mL)) and TCID50 per mL. Reduction in infection titers in plaque-forming units per mL (PFU/mL) for control and calcium alginate films (AlgCa) at 30 min of viral contact. *** *p* > 0.001. Student’s *t*-test (*** *p* > 0.001).

The synthesized calcium alginate films showed also antiviral activity against SARS-CoV-2 Delta variant with 1.64-log reduction viral infectivity reduction (96.94% viral inactivation) after 30 min of contact (Figure 5).

This reduction was even slightly greater than that observed with bacteriophage phi 6 (Figure 3). The antiviral mechanism of calcium alginate is not clearly understood yet [9]. Thus, a study of alginic acid showed that this anionic biopolymer can bind to a viral envelope, such as that of the rabies virus, inactivating its replication [41]. In this context, several studies have attributed the antiviral action of alginate to the aggregation of viral particles in solution [24,42]. Moreover, an antiviral study using guluronic acid-rich sodium alginate against Herpes simplex virus type 1 (HSV-1) showed that the antiviral capacity of this biopolymer could be associated with the direct interference with the herpes virion enveloped structures associated with cell adsorption [43], in good agreement with other compounds such as monoterpenes derived from essential oils [44] or *Pelargonium sidoides* extracts [45]. A study performed with sodium alginate showed strong antiviral activity against the enveloped HSV-1 viral particles [9]. However, another study showed only slight viral inhibition (15–20%) of alginic acid against the enveloped rubella virus (RV) [46]. Calcium alginate in the form of fibers or microcapsules have shown previously low or negligible antiviral activity (≤0.6-log reduction in virus titers or 75% viral inactivation) against the enveloped IFV [25], HCV, and SINV [26] RNA viruses. However, in this work, the antiviral properties of calcium alginate were studied in the form of thin films. Therefore, the increase in antiviral activity (Figure 3 and Figure 5) in this form of calcium alginate with respect to those previous studies can be attributed to the increase in concentrated negative charges. Thus, the synthetic procedure followed in this study dramatically compacted the alginate polymer chains with negative charge by solvent casting to form dry thin films of sodium alginate that were subsequently crosslinked with CaCl_2_.

On the other hand, calcium alginate in the form of films has shown low or negligible antiviral activity (0.42-log reduction in virus titers or 38.23% viral inactivation) against the non-enveloped double-stranded DNA bacteriophage T4, even after 48 h of viral contact [47]. However, it is important to mention that non-enveloped viruses are more resistant to inactivation than enveloped viruses [48].

**Figure 6:**
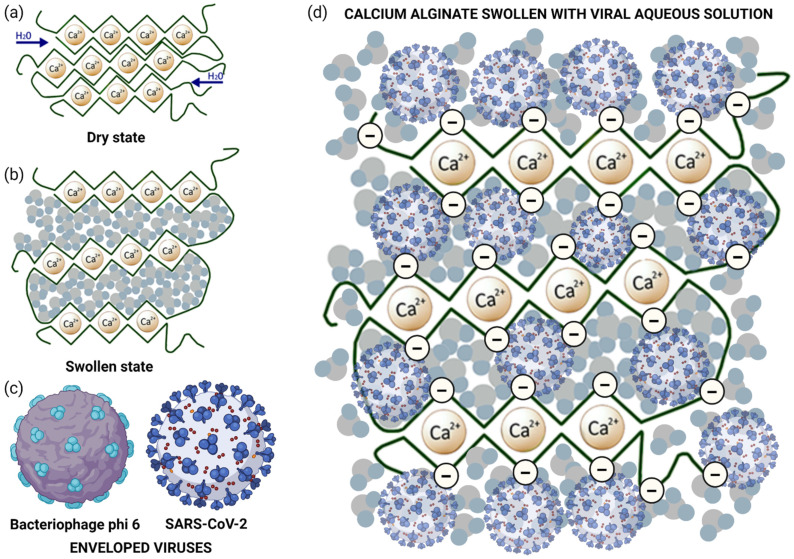
Inactivation mechanism of SARS-CoV-2 Delta variant and bacteriophage phi 6, in the synthesized negatively charged calcium alginate film: (**a**) calcium alginate structure in dry state according to the egg-box model Reprinted with permission under a Creative Commons CC BY License from ref. [21]. Copyright 2017 Spinger Nature; (**b**) calcium alginate in swollen state after being in contact with a viral aqueous solution Reprinted with permission under a Creative Commons CC BY License from ref. [21]. Copyright 2017 Spinger Nature; (**c**) enveloped RNA viruses: bacteriophage phi 6 and SARS-CoV-2 viral morphologies. Created by Ángel Serrano-Aroca with Biorender.com; and (**d**) negatively-charged calcium alginate interfering with SARS-CoV-2 Delta variant in viral aqueous solution. Created by Ángel Serrano-Aroca with Biorender.com.

Therefore, the low or strong antiviral activity of calcium alginate hydrogels could be associated with the total and proximity of negative charges of the biopolymer network. The hydrophilic calcium alginate films swell when they are in contact with a viral aqueous solution [21] as depicted in Figure 6a,b.

Thus, the two types of viral particles (Figure 6c) can interact with the negatively charged calcium alginate film surface or porous structure as depicted in Figure 6d. These negative charges can bind to the enveloped RNA viruses, inactivating their capacity of infection by interaction with viral structures required for cell adsorption and thus inhibiting viral entry to cells. Therefore, in this study, we have developed biocompatible calcium alginate films with antiviral activity against two enveloped viruses. These results show great promise to combat SARS-CoV-2 and other enveloped viruses in the current and future pandemics.

## 4. Conclusions

Biomaterial films of calcium alginate have shown antiviral activity (≥94.92% of viral inactivation) against two enveloped viruses, bacteriophage phi 6 (94.92% viral inactivation) and SARS-CoV-2 Delta variant (96.94% viral inactivation), and no cytotoxic effects in human keratinocytes for the first time in the literature. The antiviral mechanism can be attributed to the negatively charged biopolymer network of calcium alginate that can bind to viral envelopes, inactivating its replication. These anti-bacteriophage and anti-SARS-CoV-2 biocompatible films of calcium alginate are very promising for a broad range of industrial applications.

## Data Availability

Not applicable.
